# The cystic fibrosis treatment Trikafta affects the growth, viability, and cell wall of *Aspergillus fumigatus* biofilms

**DOI:** 10.1128/mbio.01516-23

**Published:** 2023-10-13

**Authors:** Jane T. Jones, Kaesi A. Morelli, Elisa M. Vesely, Charles T. S. Puerner, Chetan K. Pavuluri, Brandon S. Ross, Norman van Rhijn, Michael J. Bromley, Robert A. Cramer

**Affiliations:** 1 Department of Microbiology and Immunology, Dartmouth Geisel School of Medicine, Hanover, New Hampshire, USA; 2 Manchester Fungal Infection Group, Division of Evolution, Infection, and Genomics, Faculty of Biology, Medicine and Health, University of Manchester, Manchester, United Kingdom; 3 Antimicrobial Resistance Network, University of Manchester, Manchester, United Kingdom; Duke University Hospital, Durham, North Carolina, USA

**Keywords:** *Aspergillus fumigatus*, Trikafta, cystic fibrosis, anti-fungal agents, drug susceptibility

## Abstract

People with cystic fibrosis (pwCF) commonly test positive for the pathogenic fungus *Aspergillus fumigatus*, which is associated with a decline in lung function. Trikafta is a recently approved therapy for pwCF that improves quantity and function of the CFTR protein; however, it is not known how Trikafta affects microbial communities in the lung. Therefore, the aim of this study was to determine whether Trikafta directly affects *A. fumigatus* growth and biology. While Trikafta did not impact the viability of *A. fumigatus* conidia, treatment of *A. fumigatus* biofilms with Trikafta reduced overall biofilm biomass. This finding was associated with increased membrane permeability, decreased viability, and reduced metabolic activity following long-term treatment of biofilms. Trikafta-induced membrane permeability, and biomass reduction was partially blocked with the calcium channel inhibitor verapamil and fully blocked by the mammalian CFTR inhibitor GlyH-101. Trikafta-induced biomass reduction and metabolic activity was shown to be regulated by the high-osmolarity glycerol pathway gene *sakA*. Trikafta treatment also induced resistance to the cell wall stressor calcofluor white, susceptibility to the anti-fungal drug caspofungin, and decreased inflammatory responses from murine bone marrow cells. Collectively, these results reveal that Trikafta affects infection-relevant *A. fumigatus* biology and host-microbial interactions.

**IMPORTANCE:**

PwCF commonly test positive for pathogenic fungi, and more than 90% of the cystic fibrosis patient population is approved for the modulator treatment, Trikafta. Therefore, it is critical to understand how fungal communities, specifically *A. fumigatus*, respond to Trikafta exposure. Therefore, we sought to determine whether Trikafta impacted the biology of *A. fumigatus* biofilms. Our data demonstrate that Trikafta reduces biomass in several laboratory strains as well as clinical strains isolated from the expectorated sputum of pwCF. Furthermore, Trikafta reduces fungal viability and the capacity of biofilms to recover following treatment. Of particular importance, Trikafta affects how *A. fumigatus* biofilms respond to cell wall stressors, suggesting that Trikafta modulates components of the cell wall. Since the cell wall directly affects how a host immune system will respond to and effectively neutralize pathogens, our work, demonstrating that Trikafta impacts the *A. fumigatus* cell wall, is potentially highly relevant to fungal-induced disease pathogenesis.

## INTRODUCTION

The triple drug combination therapy Trikafta (elexacaftor/tezacaftor/ivacaftor) has been approved for cystic fibrosis (CF) patients harboring one or two copies of the dF508 *cftr* allele. The combination of two molecules involved in refining CFTR protein folding (elexacaftor/tezacaftor) and another molecule to potentiate CFTR ion gating function (ivacaftor) has improved lung function, sweat chloride levels, and overall quality of life in people with cystic fibrosis (pwCF) (1, 2). Recent data have also shown that elexacaftor can improve CFTR gating function in addition to folding (3). With the approval of Trikafta use in over 90% of pwCF, it is critical to understand the long-term impact of these combination treatments on the host and the associated microbial communities in the CF lung environment. Due to a thick buildup of sticky mucus, conventional host mechanisms that prevent microbial infection are perturbed in the CF lung and lead to chronic microbial colonization and infection. A wide variety of bacterial genera have been recovered from the CF lung, including *Pseudomonas*, *Streptococcus*, *Prevotella*, and many others. Additionally, diverse fungi (including *Candida* spp. and *Aspergillus* spp.) and viruses (influenza and respiratory syncytial viruses) have also been routinely identified [reviewed in reference (4)].

The role of fungi in CF lung disease progression remains ill-defined. Due to its frequent isolation from sputum samples of pwCF and its known role as a human fungal pathogen, the impact of *A. fumigatus* within the CF lung and subsequent pathogenesis are of particular importance. Invasive infections caused by *A. fumigatus* spp. typically occur in individuals with compromised immune systems, such as in patients undergoing chemotherapy or therapy with newer immune modulating biologics (5 – 9). After *A. fumigatus* conidia germinate in the lung environment and form hyphae, a biofilm that is resistant to anti-fungal therapy develops (10, 11) that contributes to morbidity and mortality (12 – 15). However, despite a functional immune system, *A. fumigatus* is detected in lung sputum samples of approximately 40%–50% of pwCF, and *A. fumigatus* presence in the lung has been found to be associated with a sharper decline in lung function (16 – 18) compared to pwCF who test negative for *A. fumigatus*. Ivacaftor treatment is correlated with reduced *Aspergillus* species in the lungs of pwCF (19, 20), and a more recent publication showed that Orkambi (ivacaftor/lumacaftor) reduced phagocyte-induced reactive oxygen species production induced by *A. fumigatus* (21). However, there have been no studies published directly addressing the potential impact of Trikafta on the biology of *A. fumigatus* itself and the potential consequences for the CF lung environment.

The overall goal of our study was to determine the impact of Trikafta on the biology of *A. fumigatus* and whether Trikafta treatment alters the host response to this important CF-associated fungus. We observed that Trikafta treatment has no minimum inhibitory concentration (MIC) against *A. fumigatus* conidia but does have a striking impact on biofilms from several laboratory and clinical isolates. Furthermore, our studies revealed that the effects of Trikafta on *A. fumigatus* biofilms are significantly exacerbated in combination with ionic stress. Collectively, we observed that Trikafta reduces the viability of *A. fumigatus* biofilms, increases fungal membrane permeability, alters susceptibility to anti-fungal cell wall agents, and reduces the inflammatory response of murine bone marrow cells to *A. fumigatus*.

## RESULTS

### Trikafta reduces growth of both laboratory and clinical *A. fumigatus* biofilms

**Fig 1:**
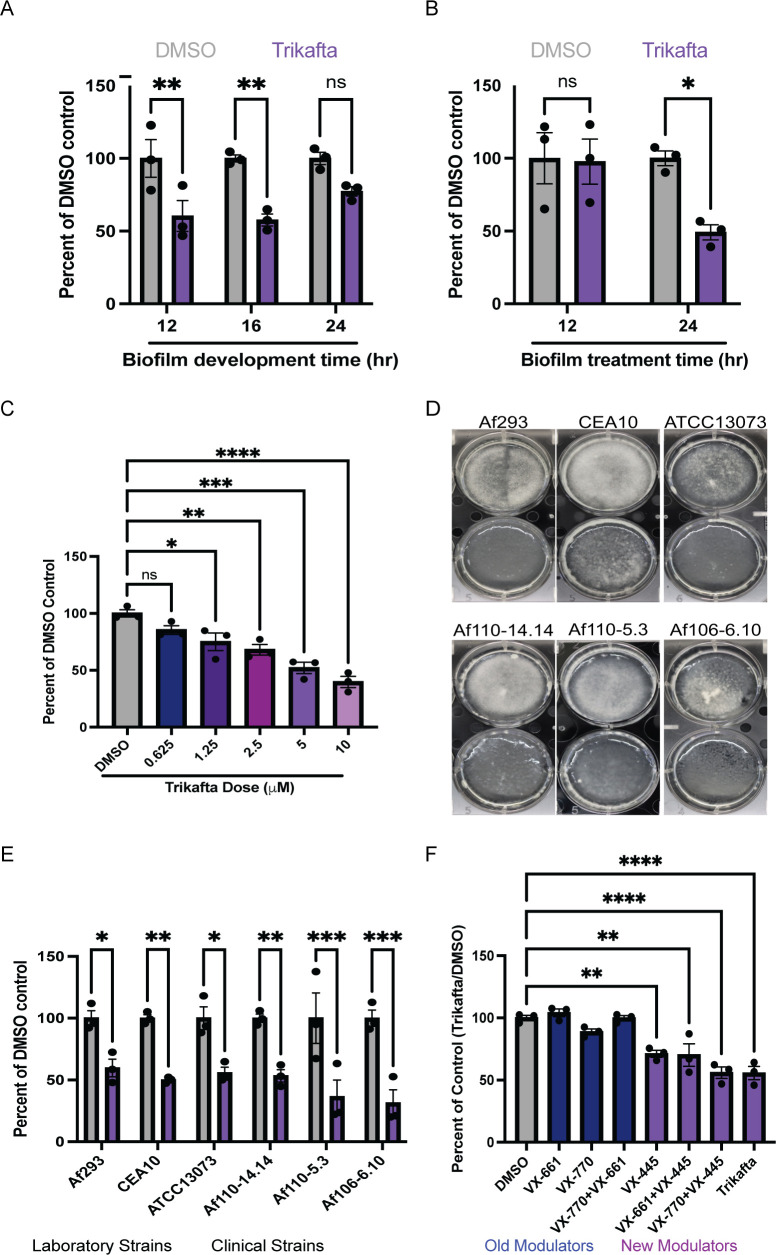
Trikafta reduces growth of both laboratory and clinical *A. fumigatus* biofilms. (**A**) CEA10 biofilms were grown in liquid glucose minimal medium (L-GMM) for 12, 16, or 24 hours. Media were removed and then fresh media with either DMSO (0.15%) or Trikafta (5 µM/molecule) were added for an additional 24 hours. Samples were collected, washed in water, and lyophilized. Dry biomass was measured, and all groups are represented as percentage of DMSO-treated average. (**B**) CEA10 biofilms were grown in L-GMM for 16 hours. Media were removed and then fresh media with either DMSO (0.15%) or Trikafta (5 µM/molecule) were added for an additional 12 or 24 hours. Samples were processed and graphically represented as in panel **A**. (**C**) CEA10 biofilms were grown in L-GMM for 16 hours. Media were removed, and then fresh media with either DMSO or the indicated dose of Trikafta (μM/molecule) were added for an additional 24 hours. Samples were processed and graphically represented as in panel A. (**D**) Biofilms of the indicated laboratory strains and clinical isolates were grown in L-GMM for 16 hours. Media were removed and then fresh media with either DMSO (0.15%) or Trikafta (5 µM/molecule) were added for an additional 24 hours, and images were taken. (**E**) Samples were processed and graphically represented as in panel **A**. (**F**) CEA10 biofilms were grown in L-GMM for 16 hours. Media were removed and then fresh media with either DMSO (0.15%), individual molecules, double combinations, or Trikafta (5 µM/molecule) were added for an additional 24 hours. Samples were processed and graphically represented as in panel **A**. For panels **A, B, and E**, a two-way analysis of variance (ANOVA) with Sidak’s multiple comparisons was used, and for panels **C and F**, a one-way ANOVA with Dunnett’s multiple comparisons with all groups compared to DMSO was used. Data points are three biological replicates, which represent the average of technical replicates. ns, not significant.

To determine whether Trikafta directly affects *A. fumigatus* growth and/or function, we first tested whether Trikafta treatment affected conidial growth. Conidia from the two most utilized laboratory reference strains, CEA10 and Af293, were grown in either standard CLSI RPMI-1640 with MOPS [3-(N-morpholino) propanesulfonic acid] buffer (22) or glucose minimal medium (GMM) with Trikafta doses ranging from 50.0 to 1.56 µM/individual modulator. The anti-fungal voriconazole was used as a positive control. While the MIC value of both strains to voriconazole was 0.25 µg/mL, consistent with previously published data, Trikafta did not affect fungal growth at any of the concentrations tested. We next tested whether Trikafta affected growth of *A. fumigatus* biofilms, which contain hyphae, at different levels of maturity. Initiating (12 hours), immature (16 hours), and mature (24 hours) CEA10 submerged biofilms were treated with either GMM plus dimethyl sulfoxide (DMSO) (vehicle control, 0.15%) or Trikafta (5 µM/molecule in every assay from this point forward) for an additional 24 hours before biomass was measured. Trikafta treatment of all stages of biofilm development reduced the total biomass, although only significantly in 12- and 16-hour biofilms (Fig. 1A). Since biomass reduction was most pronounced at 16 hours, 16-hour biofilms were treated with either GMM plus DMSO control or Trikafta for 12 and 24 hours. Surprisingly, biomass reduction occurred in the Trikafta-treated biofilms compared to DMSO-treated controls only after 24-hour treatment (Fig. 1B). We next tested a range of Trikafta doses on biomass reduction. We performed twofold serial dilutions of Trikafta (20.0–0.625 μM) on 16-hour biofilms and observed a smooth dose-response reduction in biofilm biomass, with 5 µM resulting in an approximate 50% reduction (Fig. 1C). To determine whether Trikafta effectively reduces biomass across *A. fumigatus* strains, we tested a selection of common laboratory strains (CEA10, Af293, and ATCC13073) as well as clinical isolates from pwCF respiratory samples (Af110-14.14, Af110-5.3, and Af106-6.10). After 16 hours of biofilm development, media were removed and biofilms were treated with GMM plus DMSO or Trikafta for an additional 24 hours. Biofilms were imaged and dry biomass was quantified (Fig. 1D and E). For all strains tested, Trikafta significantly reduced total fungal biomass between 50% and 70%. Strikingly, the Trikafta-treated biofilms macroscopically looked translucent and viscous, suggesting possible cell lysis (Fig. 1D).

Next we sought to define which molecule or combination of ivacaftor (VX-770), tezacaftor (VX-661), and elexacaftor (VX-445) caused the reduction in fungal biomass. CEA10 16-hour biofilms were treated with GMM plus DMSO, individual molecules, or different combinations of the individual molecules for an additional 24 hours before dry biomass was quantified (Fig. 1F). Single-molecule elexacaftor (VX-445) alone induced a significant reduction in total biomass (Fig. 1F). Interestingly, the elexacaftor/ivacaftor combination was as effective at reducing biomass as triple combination Trikafta, suggesting a potentially small but not significant effect for ivacaftor as well. Collectively, these data show that Trikafta significantly affects growth of established biofilms, primarily through the action of the dual corrector/potentiator, elexacaftor (VX-445).

### Trikafta reduces viability and increases membrane permeability of *A. fumigatus* biofilms

**Fig 2:**
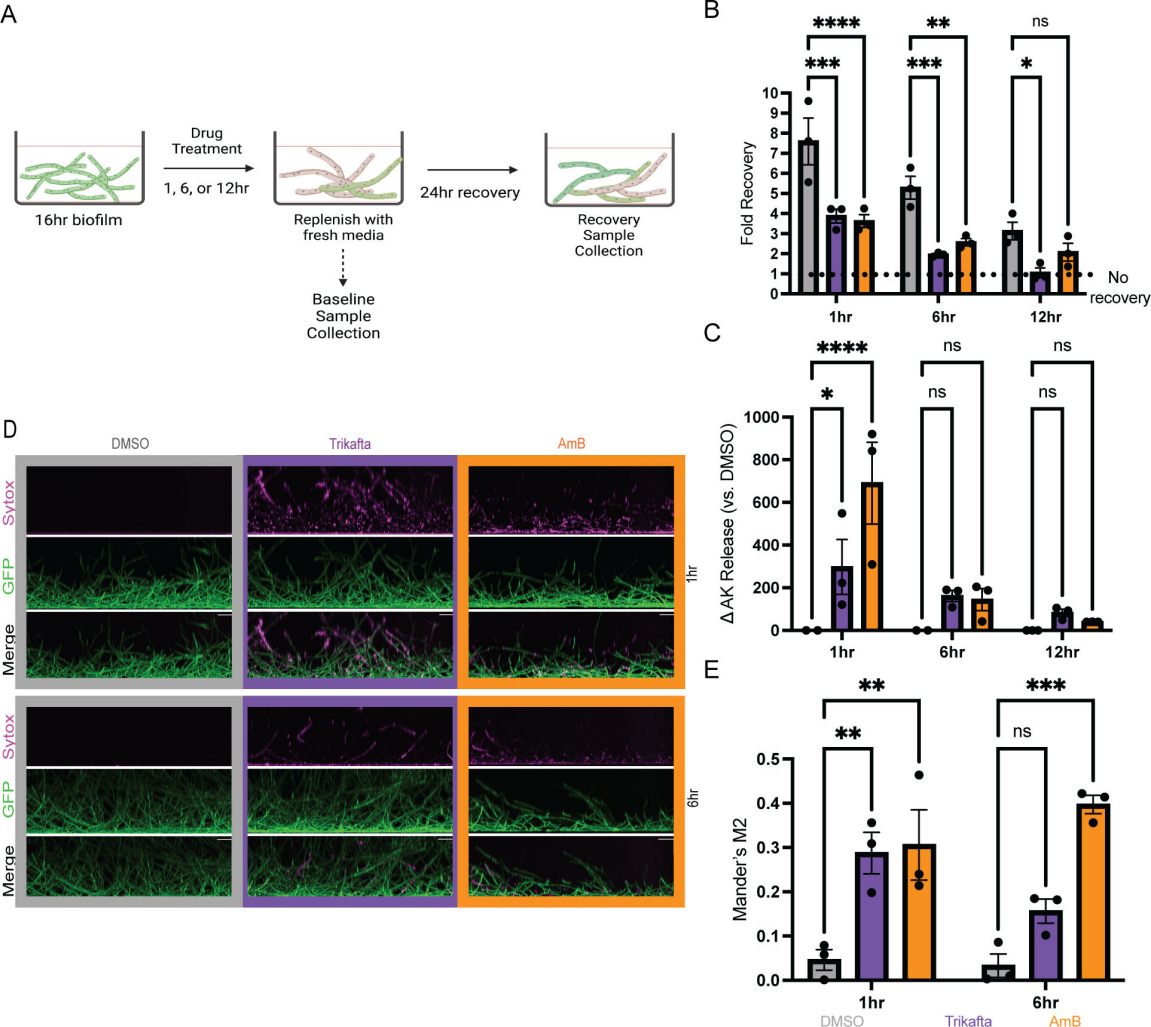
Trikafta reduces viability and increases membrane permeability of *A. fumigatus* biofilms. (**A**) Experimental design for viability assay (**B**) CEA10 biofilms were grown in L-GMM for 16 hours. Media were removed and then fresh media with either DMSO (0.15%), Trikafta (5 µM/molecule), or amphotericin B (AmB) (1 µg/mL) were added for an additional 1, 6, or 12 hours. Baseline samples were collected, and parallel biofilms had media removed and then replaced with fresh L-GMM for an additional 24 hours. Recovery biofilms were collected, washed in water, and lyophilized, and dry biomass was measured. Treatment groups were compared to their own controls, and data are represented as fold change from baseline control. (**C**) CEA10 biofilms were grown in L-GMM for 16 hours. Media were removed and then fresh media with either DMSO (0.15%), Trikafta (5 µM/molecule), or AmB (1 µg/mL) was added for an additional 1, 6, or 12 hours. Supernatant was collected and adenylate kinase (AK) was measured. Data are shown as percent change over DMSO controls (DMSO set to 100%) and then subtracting 100 to get a delta in adenylate kinase release. (**D**) CEA10-*gpdA:GFP* biofilms were grown in L-GMM for 16 hours. Media were removed and then fresh media with either DMSO (0.15%), Trikafta (5 µM/molecule), or AmB (1 µg/mL) were added for an additional 1 or 6 hours. Sytox Blue 1:1,000 was added for 5 minutes. Images were taken on Nikon spinning Disc confocal microscope ×20. (**E**) Images were quantified and represented as the fraction of GFP that overlaps with Sytox in each image (Mander’s M2). For (**B, C, and E**), a two-way ANOVA with Dunnett’s multiple comparisons was used, with both treatment groups compared to DMSO controls. Data points are three biological replicates, which represent the average of technical replicates.

Given the macroscopic observations of Trikafta-treated biofilms and the substantial loss of biomass, we sought to determine if Trikafta treatment reduced viability of the biofilms. To test this hypothesis, we determined whether Trikafta-treated biofilms could recover growth in fresh media. CEA10 biofilms were grown for 16 hours and subsequently treated with GMM supplemented with either DMSO, Trikafta, or the anti-fungal drug amphotericin B (AmB, 1 µg/mL) as a positive control for an additional 1, 6, and 12 hours. At each time point, baseline samples were collected, and in parallel, a recovery sample was prepared where media were removed and replaced with fresh GMM for a further 24 hours of incubation before dry biomass weight was quantified (Fig. 2A). Biofilms treated with Trikafta and AmB for 1 and 6 hours had around 50% of the recovery of DMSO-treated biofilms. After 12-hour treatment with Trikafta, biofilms had no recovery compared to threefold recovery of DMSO-treated biofilms (Fig. 2B).

Next, we determined whether Trikafta treatment affected overall membrane permeability of *A. fumigatus* biofilms. Supernatant from either DMSO or Trikafta-treated biofilms for 1, 6, or 12 hours was analyzed for adenylate kinase (AK) activity, a marker of membrane permeability and damage (23). Treatment with Trikafta or AmB for 1 hour significantly increased the change in AK release versus DMSO-treated controls approximately 300% and 600%, respectively. After 6- and 12-hour treatment with Trikafta and AmB, detectable levels of AK were observed to around 50%–100% change from DMSO controls, although to a much lesser extent than 1 hour treatment (Fig. 2C). To further validate this observation, the cell viability stain, Sytox Blue, was used to image damage to biofilms. The stain was used in conjunction with a fluorescently labeled *A. fumigatus* strain constitutively expressing green fluorescent protein (GFP) in the cytoplasm. The GFP fluorescence allowed for identifying the overlap in Sytox signal with hyphal cells in the biofilm. CEA10-*gpdA:GFP* conidia were grown into 16 hour biofilms and treated with GMM plus either DMSO, Trikafta, or Amphotericin B for an additional 1 and 6 hours prior to staining with Sytox Blue (12 hours treatment was too long for interpretable microscopy images). Biofilms treated with GMM plus DMSO showed no intracellular Sytox Blue staining while biofilms treated with AmB showed around 30–40% positive staining at both 1 and 6 hour treatments (Fig. 2D and E). Biofilms treated with Trikafta for 1 hour were also around 30% positive for Sytox Blue and around 15% positive after 6 hours of treatment (Fig. 2D and E). Collectively, these data demonstrate that Trikafta increases membrane permeability and reduces the viability of *A. fumigatus* biofilms.

### Trikafta modulates ion channels in *A. fumigatus* biofilms

**Fig 3:**
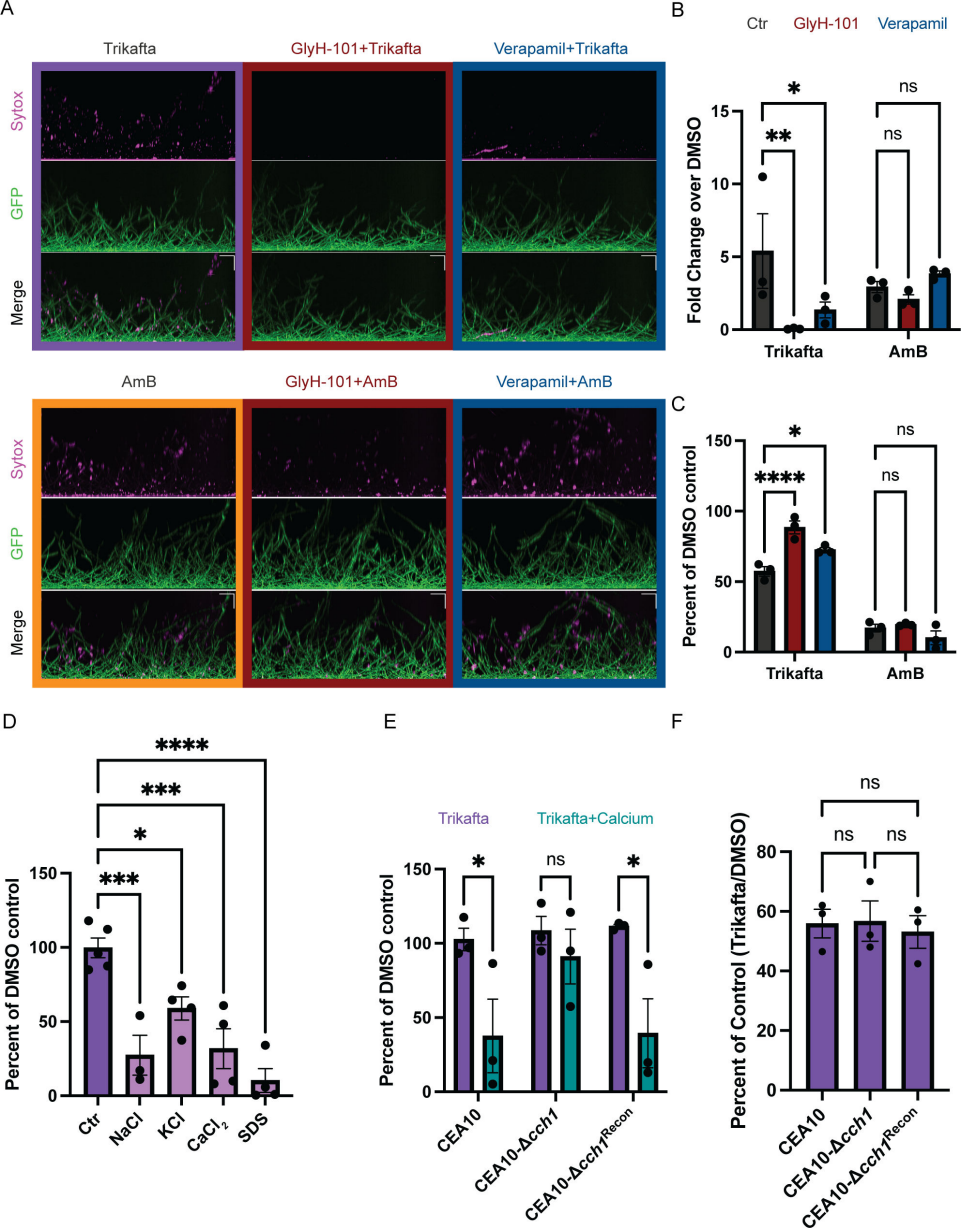
Trikafta modulates ion channels in *A. fumigatus* biofilms. (**A**) CEA10-*gpdA:GFP* biofilms were grown in L-GMM for 16 hours. Media were removed and biofilms were treated with DMSO (control, 0.2%), verapamil (calcium channel inhibitor, 1 mM), or GlyH-101 (CFTR inhibitor, 20 µM) for 1 hour. Media with either DMSO (0.15%), Trikafta (final concentration 5 µM/molecule), or AmB (final concentration 1 µg/mL) was added for an additional 1 hour. Sytox Blue 1:1,000 was added for 5 minutes. Images were taken on Nikon spinning disc confocal microscope at ×20. (**B**) Images were quantified and represented as the fraction of GFP that overlaps with Sytox in each image (Mander’s M2), and data are represented as fold change over DMSO-treated control. (**C**) CEA10 biofilms were grown for 16 hours and media were removed. Fresh media with either DMSO (0.2%), GlyH-101 (20 µM), or verapamil (1 mM) was added for 1 hour. Subsequently, media with either DMSO (0.15%), AmB (final dose 1 µg/mL), or Trikafta (final dose 5 µM/molecule) was added for an additional 24 hours. Samples were processed and graphically represented as in Fig. 1A. (**D**) CEA10 biofilms were grown in L-GMM for 16 hours. Media were removed, and then fresh media with either DMSO (0.15%) or Trikafta (5 µM/molecule) were added in either vehicle control (**H_2_0**) or 0.5M NaCl, CaCl_2_, or KCl or 0.002% SDS for an additional hour. Metabolic activity was measured using XTT assay. Data are normalized as percentage of Trikafta/DMSO controls in each condition. (**E**) Wild-type (WT) CEA10, CEA10-Δ*cch1*, and CEA10-*cch1^rec^* biofilms were grown in L-GMM for 16 hours. Media were removed and then fresh media with either DMSO (0.15%) or Trikafta (5 µM/molecule) was added in either control or 0.5-M CaCl_2_ for an additional hour, and an XTT assay was performed. Data are normalized as percentage of Trikafta/DMSO controls in either vehicle control (H_2_O) or CaCl_2_. (**F**) WT CEA10, CEA10-Δ*cch1*, and CEA10-*cch1^rec^* biofilms were grown in L-GMM for 16 hours. Media were removed, and then fresh media with either DMSO (0.15%) or Trikafta (5 µM/molecule) were added for an additional 24 hours. Samples were processed and graphically represented as in Fig. 1A. For B, C, and E, a two-way ANOVA with Dunnett’s (**B and C**) or Sidak’s (**E**) multiple comparisons was performed and a one-way ANOVA (**D and F**) with Dunnett’s (**D**) or Tukey’s (**F**) multiple comparisons was performed. Data points are three biological replicates, which represent the average of technical replicates.

Trikafta modulates ion channel function in mammalian cells, so we next hypothesized that Trikafta-induced modulation of fungal ion channels causes membrane disruption and increased membrane permeability. We used a mammalian CFTR inhibitor GlyH-101 (24) and the calcium channel inhibitor verapamil to address this hypothesis. CEA10-*gpdA:GFP* conidia were grown into 16-hour biofilms and treated with either DMSO control, GlyH-101 (20 µM), or verapamil (1 mM) for 1 hour and then subsequently treated with Trikafta for an additional 1 hour prior to Sytox Blue membrane permeability staining. Pre-treatment of biofilms with GlyH-101 or verapamil prior to Trikafta treatment almost completely abolished Trikafta-induced Sytox Blue staining (Fig. 3A and B). To test if this was specific to Trikafta, we performed the same set of experiments with amphotericin B. Pre-treatment with GlyH-101 or verapamil had little to no effect on amphotericin B-induced Sytox staining (Fig. 3A and B). To determine whether ion channel modulation contributes to Trikafta-induced reduction in biomass, 16-hour biofilms were pre-treated with GlyH-101 or verapamil for 1 hour then treated with Trikafta or amphotericin B for 24 hours. GlyH-101 almost fully rescued the biomass reduction of Trikafta, while verapamil partially rescued it (Fig. 3C). GlyH-101 and verapamil had no effect on amphotericin B-induced biomass reduction (Fig. 3C). Therefore, the impact of ion channel inhibitors on Trikafta is likely specific to its mechanism of action, since the inhibitors did not affect AmB-induced membrane damage.

We next determined whether the effects of Trikafta were exacerbated in the presence of ionic stress. We chose to utilize a 2,3-bis-(2-methoxy-4-nitro-5-sulfophenyl)-2H-tetrazolium-5-carboxanilide (XTT)-based metabolic assay to measure the metabolic activity of Trikafta-treated biofilms after 1-hour treatment because there was little to no effect of Trikafta on metabolic activity at that time point (Fig. 3D). Therefore, we treated 16-hour biofilms with either GMM plus DMSO control or Trikafta for 1 hour in either control, 0.5-M NaCl, 0.5-M CaCl_2_, or 0.5-M KCl. In parallel, we also included 0.002% SDS as a control for general membrane disruption. It is important to note that the low dose of SDS used did not cause any reduction in metabolic activity on its own in contrast to the different ions (data not shown). Strikingly, Trikafta treatment reduced metabolic activity by more than 70% in the presence of NaCl, CaCl_2_, and SDS. Trikafta also reduced metabolic activity by more than 40% in the presence of KCl (Fig. 3D). We next hypothesized that a strain harboring a null mutant in the calcium channel gene, *cch1* (25), would be resistant to Trikafta-reduced metabolic activity in CaCl_2_ stress conditions. CEA10-Δ*cch1* biofilms treated with Trikafta in the presence of CaCl_2_ were resistant to reduced metabolic activity compared to wild-type (WT) CEA10 biofilms (Fig. 3E). However, the CEA10-Δ*cch1* biofilms treated with Trikafta for 24 hours had roughly equal susceptibility to Trikafta as WT controls, indicating that exogenous CaCl_2_ levels may impact Trikafta’s antifungal effects (Fig. 3E). Taken together, these data suggest that the antifungal effects of Trikafta are exacerbated in ionic stress conditions.

### Trikafta transiently increases metabolic activity in *A. fumigatus* biofilms

**Fig 4:**
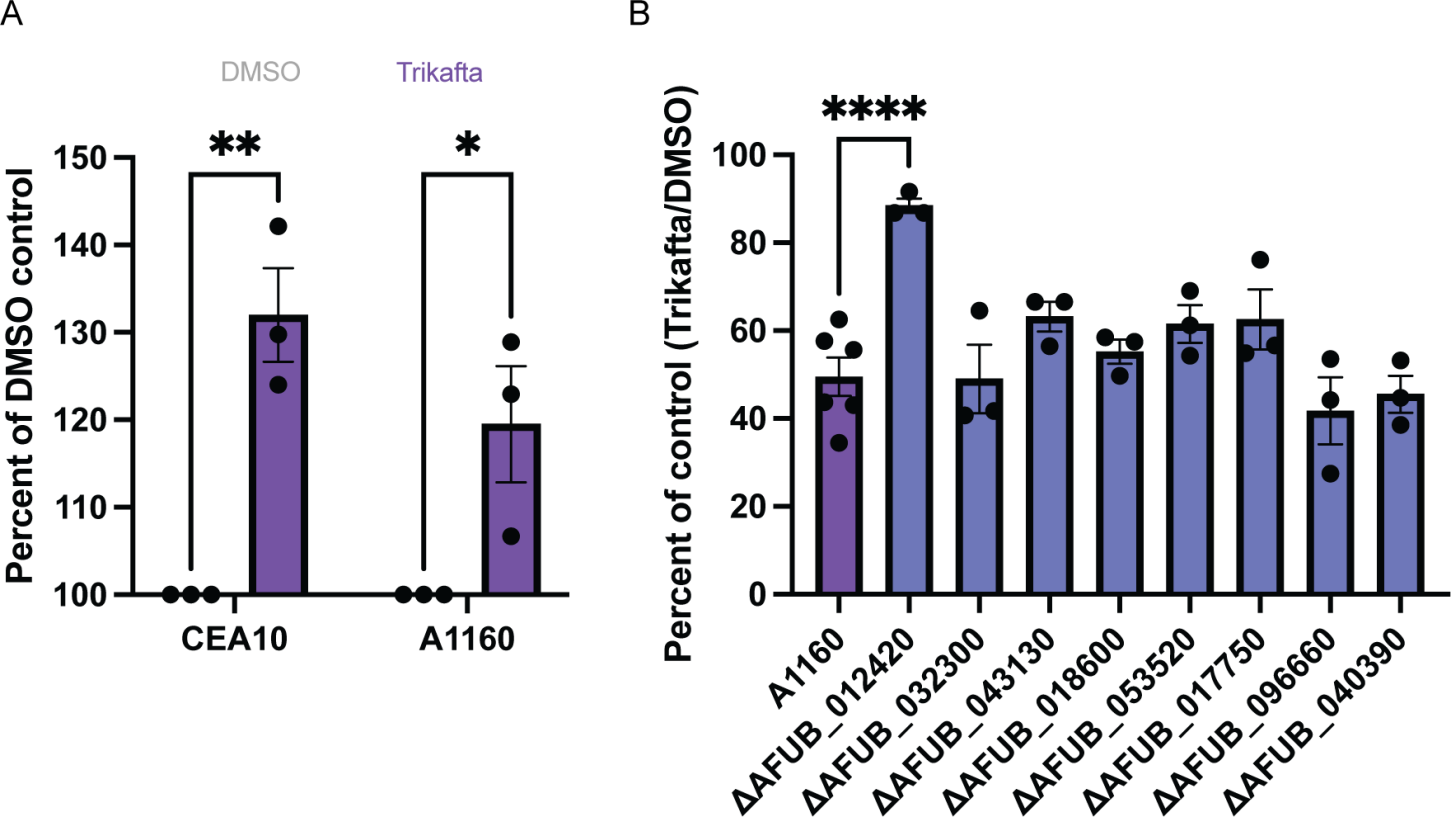
Trikafta acutely increases metabolic activity in *A. fumigatus* biofilms. (**A**) CEA10 or A1160 biofilms were grown in L-GMM for 16 hours. Media were removed, and then fresh media with either DMSO (0.15%) or Trikafta (5 µM/molecule) were added for an additional 30 min. The metabolic dye, resazurin, was added 10% vol for an additional 5 hours after Trikafta treatment and fluorescence 594 was captured. A two-way ANOVA with Sidak’s multiple comparisons was used. Data points are three biological replicates, which represent the average of technical replicates. (**B**) Biofilms of the indicated kinase library mutant strains were grown in L-GMM for 16 hours. Media were removed, and then fresh media with either DMSO (0.15%) or Trikafta (5 µM/molecule) were added for an additional 24 hours. Samples were processed and graphically represented as in Fig. 1A. Three to six technical replicates are represented.

Since the CEA10-Δ*cch1* mutant biofilm was not resistant to Trikafta-induced biomass reduction, we further sought to determine how Trikafta reduced biofilm biomass after long-term exposure to the drug. To establish an assay for further exploring the metabolic changes in fungal biomass, we evaluated biofilms over time using the metabolic dye resazurin, as it allows for data collection over a range of time rather than a snapshot in time as with XTT-based metabolic assays. We treated 16-hour biofilms with either GMM plus DMSO or Trikafta for 30 minutes and then added resazurin for an additional 5 hours. Surprisingly, analysis of metabolic activity in Trikafta-treated biofilms compared to DMSO-treated controls showed a 30%–40% increase in metabolic activity in CEA10 biofilms (Fig. 4A). We hypothesized that this increased metabolic activity was likely due to increased mitochondrial activity.

To begin to delineate pathways by which Trikafta acts on *A. fumigatus* biofilm biomass reduction, we used this resazurin assay to perform a genetic screen on an *A. fumigatus* kinase null mutant library. Importantly, we observed a similar increase in metabolic activity in the WT A1160 background strain used to generate the null mutant library upon Trikafta treatment (Fig. 4A). A total of 108 putative kinase null mutants (Table 1) were grown into 16-hour biofilms, treated with either GMM plus DMSO or Trikafta for 30 minutes, and subsequently treated with resazurin for an additional 5 hours. For each run, the effect of Trikafta-induced metabolic activity in the parent strain (A1160) was calculated and used as the reference. A total of 18 mutant strains had altered metabolic activity in response to Trikafta compared to the control strain, based upon being either 20% below or 15% above the control strain (Table 2). Eight mutant strains (in bold) were selected to screen for biomass phenotypes, and it was found that a strain with a null mutation in ΔAFUB_012420 [high-osmolarity glycerol (HOG) pathway kinase, *sakA*] was the least susceptible to Trikafta-induced biomass reduction (Fig. 4B).

**TABLE 1:** List of *A. fumigatus* kinase null mutant strains (A1160)

Kinase null mutants		
AFUB_045810	AFUB_006320	AFUB_096030
AFUB_021710	AFUB_052630	AFUB_096080
AFUB_053300	AFUB_035220	AFUB_099990
AFUB_012420	AFUB_019930	AFUB_006190
AFUB_032300	AFUB_017750	AFUB_089280
AFUB_044560	AFUB_011380	AFUB_101210
AFUB_030660	AFUB_027890	AFUB_044260
AFUB_052450	AFUB_056020	AFUB_035990
AFUB_043130	AFUB_059390	AFUB_096660
AFUB_010510	AFUB_059090	AFUB_063270
AFUB_018770	AFUB_066150	AFUB_020650
AFUB_020560	AFUB_071620	AFUB_045550
AFUB_029320	AFUB_056110	AFUB_060950
AFUB_025560	AFUB_082830	AFUB_061120
AFUB_038630	AFUB_077790	AFUB_040390
AFUB_029240	AFUB_075210	AFUB_081220
AFUB_016170	AFUB_090090	AFUB_076300
AFUB_048440	AFUB_078920	AFUB_075230
AFUB_006780	AFUB_066030	AFUB_074100
AFUB_039620	AFUB_095720	AFUB_089460
AFUB_045840	AFUB_060320	AFUB_093800
AFUB_051750	AFUB_087320	AFUB_067080
AFUB_029820	AFUB_079830	AFUB_017740
AFUB_027640	AFUB_078980	AFUB_100220
AFUB_038060	AFUB_053960	AFUB_036640
AFUB_001600	AFUB_070630	AFUB_009070
AFUB_053500	AFUB_087120	AFUB_096590
AFUB_018600	AFUB_075350	AFUB_045710
AFUB_010360	AFUB_054020	AFUB_030290
AFUB_044400	AFUB_081540	AFUB_001940
AFUB_027480	AFUB_055480	AFUB_095050
AFUB_030570	AFUB_056640	AFUB_047210
AFUB_053520	AFUB_074550	AFUB_063830
AFUB_039100	AFUB_071600	AFUB_101530
AFUB_007300	AFUB_098230	AFUB_071990
AFUB_014350	AFUB_099170	AFUB_080040

**TABLE 2:** Trikafta treatment alters metabolic activity in select kinase null mutants^*b*^

AFUB no.	Putative function	% change from vehicle	% change from WT
AFUB_012420	Mitogen-activated protein kinase	176.5	**139.9**
AFUB_053520	Calcium/calmodulin-dependent protein kinase, putative	156.6	**118.6**
AFUB_017750	Protein kinase, putative	154.8	**117.3**
AFUB_099170	Protein kinase Yak1, putative	129.1	112.3
AFUB_018600	Protein kinase, putative	105.4	**79.9**
AFUB_101530	Sensor histidine kinase/response regulator, putative	91.6	79.7
AFUB_098230	Inositol kinase kinase (UvsB), putative	91.0	79.1
AFUB_043130	MAP kinase kinase Ste7	99.4	**78.8**
AFUB_067080	Putative uncharacterized protein	90.1	78.4
AFUB_055480	Serine/threonine protein kinase, putative	88.2	76.7
AFUB_061120	Protein kinase domain-containing protein	87.9	76.5
AFUB_045550	Calcium/calmodulin-dependent protein kinase, putative	86.6	75.4
AFUB_020650	Two-component osmosensing histidine kinase (Bos1)	85.8	74.7
AFUB_032300	Protein kinase, putative	93.2	**73.9**
AFUB_071990	N/A^*a*^	84.6	73.6
AFUB_063830	Ubiquinone biosynthesis protein, putative	84.0	73.0
AFUB_096660	Putative uncharacterized protein	76.7	**66.7**
AFUB_040390	Protein kinase domain-containing protein	72.6	**63.2**

^*a*^ N/A, not available.

^*b*^ The values in bold were selected for a secondary biomass screen.

### Trikafta activates SakA and reduction of SakA activity increases resistance of *A. fumigatus* biofilms to Trikafta

**Fig 5:**
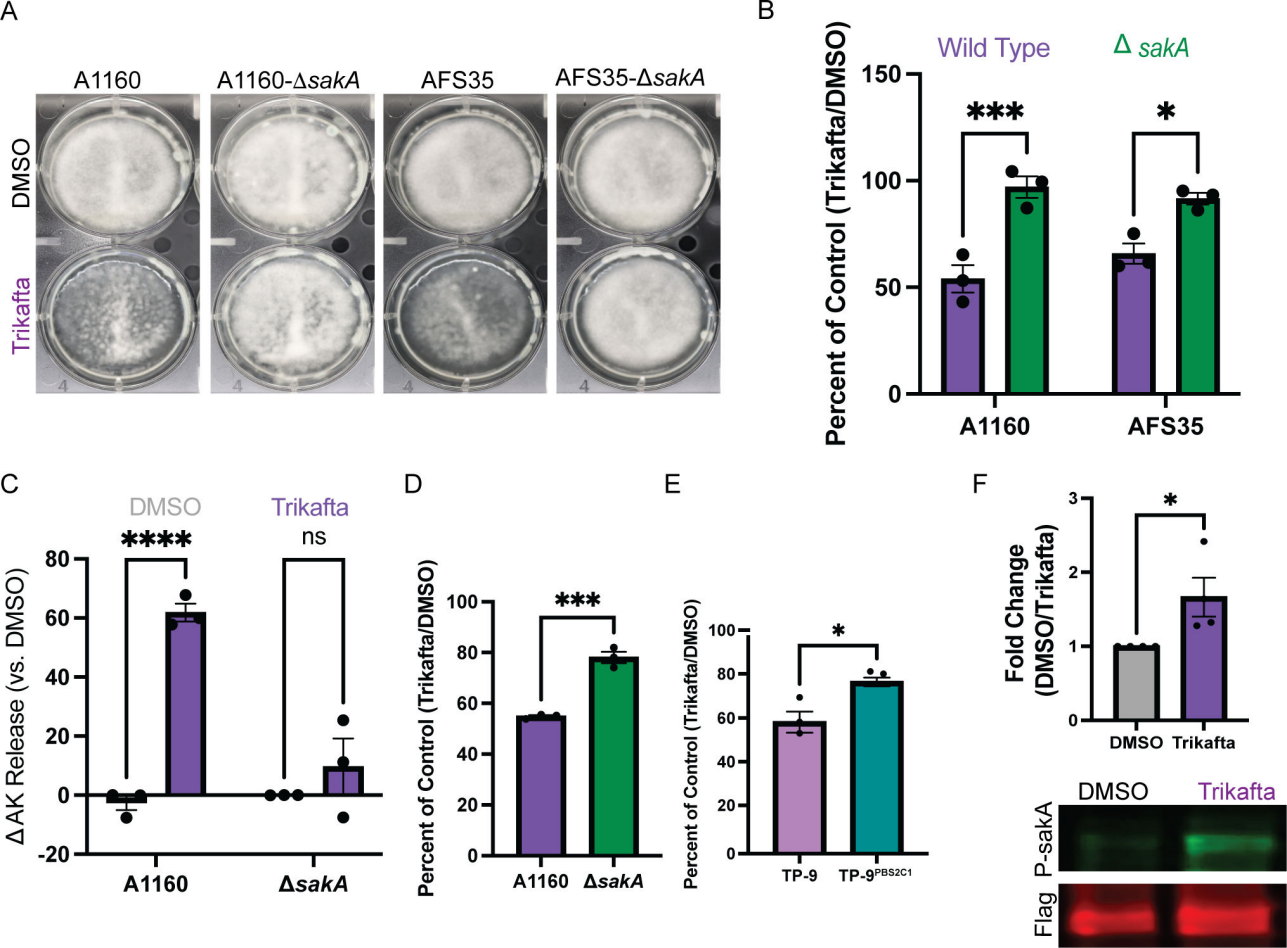
Trikafta activates SakA, and reduction of SakA activity increases resistance of *A. fumigatus* biofilms to Trikafta. (**A**) WT (A1160 and Afs35) and Δ*sakA* conidia (in both WT strains) were grown for 16 hours into biofilms. Media were removed, and then fresh media with either DMSO (0.15%) or Trikafta (5 µM/molecule) were added for an additional 24 hours and images were taken. (**B**) Samples were processed and graphically represented as in Fig. 1A. (**C**) WT A1160 and A1160-Δ*sakA* biofilms were grown in L-GMM for 16 hours. Media were removed, and then fresh media with either DMSO (0.15%) or Trikafta (5 µM/molecule) were added for an additional 12 hours. Supernatant was collected and adenylate kinase was quantified. Data are shown as percent change over DMSO controls (DMSO set to 100%) and then subtracting 100 to get a delta in AK release. (**D**) In parallel to panel** C**, metabolic activity of biofilms was measured by XTT. Data are represented as percentage of DMSO controls (Trikafta/DMSO). (**E**) TP9 and TP-9^PBS2C1^ biofilms were grown for 16 hours. Media were removed, and then fresh media with either DMSO (0.15%) or Trikafta (5 µM/molecule) were added for an additional 24 hours. Samples were processed and graphically represented as in Fig. 1A. (**F**) TP-9^PBS2C1: *sakA* Flag^ biofilms were grown in L-GMM for 16 hours. Media were removed, and then fresh media with either DMSO (0.15%) or Trikafta (5 µM/molecule) were added for an additional 30 minutes. Biofilms were collected and protein extracted for Western blot analysis of either total SakA (Flag) or P-SakA. Samples were normalized to total protein and quantified using LI-COR. A two-way ANOVA with Sidak’s multiple comparisons (**B and C**) and Student’s *t*-test (**D–F**) were performed. Data points are three biological replicates, which represent the average of technical replicates.

To confirm if loss of *sakA* alters biofilm biomass reduction to Trikafta, we next tested A1160-WT, A1160-Δ*sakA*, Afs35-WT, and Afs35-Δ*sakA* biofilms that were grown for 16 hours and treated with either DMSO or Trikafta for an additional 24 hours prior to imaging (Fig. 5A). Quantification of biomass showed that while both WT (A1160 and Afs35) biofilms had approximately 50%–60% reduction in biomass with Trikafta treatment compared to controls, both ∆*sakA* mutant biofilms had little to no reduction in biomass with Trikafta treatment (Fig. 5B). We also observed that A1160-Δ*sakA* biofilms were resistant to Trikafta-induced AK release after 12-hour treatment (Fig. 5C). To further determine the effects of Trikafta at this later time point, 16-hour A1160 and A1160-Δ*sakA* biofilms were treated with either DMSO or Trikafta for 12 hours, and metabolic activity was assessed by XTT reduction. Excitingly, Trikafta caused reduction of metabolic activity by 50% in WT A1160, while A1160-Δ*sakA* biofilms only had an approximately 20% reduction in metabolic activity (Fig. 5D). This further supports our hypothesis that the initial metabolic burst observed in response to Trikafta is followed by a marked reduction in metabolic activity and that Trikafta-induced changes in metabolic activity are regulated by *sakA*.

Previous work from our laboratory observed that an *A. fumigatus* clinical isolate from a pwCF, designated TP-9, harbors mutations in the MAP kinase kinase *pbs2* that regulates SakA pathway activity. This unique *pbs2* allele arose from long-term growth in the CF lung environment and functionally leads to increased activation of SakA as indicated by phosphorylation (26). An allele swap with a *pbs2* allele from a different *A. fumigatus* clade isolate from the same individual generated the strain TP-9^PBS2C1^ and resulted in reduced SakA activation compared to TP-9 (26). To further test the effects of SakA activity on the response to Trikafta, we next compared Trikafta-induced effects on these two strains. TP-9 and TP-9^PBS2C1^ biofilms were grown for 16 hours and treated with GMM plus either DMSO or Trikafta for an additional 24 hours. Quantification of biofilm biomass showed that while TP-9 biofilms had almost 45% reduction in biomass with Trikafta treatment compared to controls, TP-9^PBS2C1^ only had 20% reduction, further showing that a reduction in SakA activity conferred resistance to Trikafta-induced biomass reduction (Fig. 5E). Additionally, we wanted to confirm if Trikafta induced phosphorylation of SakA. Utilizing the TP-9^PBS2C1:*sakA*-Flag^ strain (26), we treated 16-hour biofilms with GMM plus either DMSO or Trikafta for 30 minutes, collected the biomass, and subjected the protein to SDS-PAGE and subsequent Western blot analysis for total SakA (Flag) and P-SakA. Trikafta treatment increased P-SakA almost twofold in comparison to DMSO-treated controls, suggesting that Trikafta treatment activates SakA in *A. fumigatus* biofilms (Fig. 5F).

### Trikafta modulates the biofilm cell wall and mediates inflammatory responses in host immune cells

**Fig 6:**
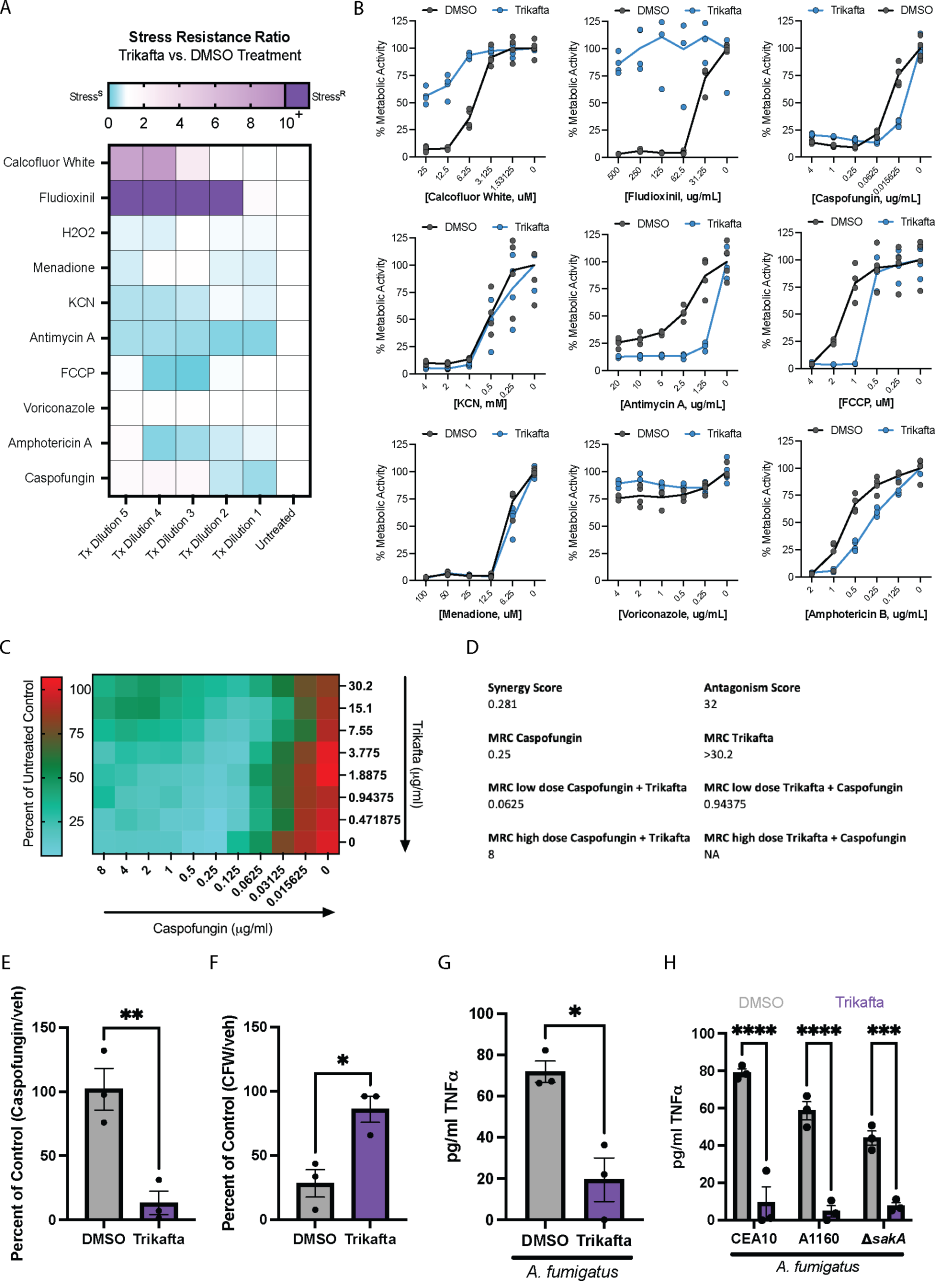
Trikafta modulates the biofilm cell wall and mediates inflammatory responses in host immune cells. (**A**) CEA10 biofilms were grown in L-GMM for 16 hours. Media were removed, and then fresh media with either DMSO (0.15%) or Trikafta (5 µM/molecule) plus serial dilutions of the indicated stressors (CFW, Fludioxonil, caspofungin, KCN, antimycin A, FCCP, menadione, voriconazole, and AmB) were added for an additional 3 hours. Metabolic activity was measured by the reduction of the dye, XTT. Stress resistance ratio (Trikafta/DMSO controls) is represented by highly resistant (purple) through highly susceptible (blue) as a consequence of Trikafta co-treatment. (**B**) Graphs represent the percentage of stressor/control in the presence or absence of Trikafta. Data represent five technical replicates. (**C**) CEA10 biofilms were grown in L-GMM for 16 hours. Media were removed and then fresh media with serial two-fold dilutions of Trikafta [30.2 µg/mL(20 µM/molecule)−0.471875 µg/mL (0.3125 µM/molecule)] in checkerboard combination with serial two-fold dilutions of caspofungin (8 µg/mL −0.015625 µg/mL). Heat map represents percent of untreated control values, with higher metabolic activity indicated by red and lower indicated by green. Data are averages of three biological replicates. (**D**) Synergy and antagonism scores for caspofungin and Trikafta based on values generated in C. (MRC = Metabolic reduction concentration). (**E**) CEA10 biofilms were grown in L-GMM for 16 hours. Media were removed and then fresh media with either DMSO (0.15%) or Trikafta (5 µM/molecule) plus 0.015625 µg/mL caspofungin or (**F**) 12.5 µM CFW for 3 hours. An XTT assay was performed to determine metabolic activity, and data are shown as percentage of stressor/control in either DMSO or Trikafta. (**G**) Bone marrow cells were isolated from C57Bl/6 mice harboring dF508 mutation in *cftr* and seeded 1 × 10^7^/mL in co-culture with CEA10 conidia (1 × 106^6^/mL) for 16 hours in the presence of interferon gamma (IFN-γ) (10 ng/mL) with either DMSO (0.15%) or Trikafta (5 µM). 96 well plates were centrifuged and supernatants were analyzed for murine tumor necrosis factor alpha (TNF-α). (**H**) Bone marrow cells were isolated from WT C57Bl/6 mice and seeded 1 × 10^7^ /mL in co-culture with CEA10, A1160, or A1160-Δ*sakA* conidia (1 × 10^6^ /mL) for 16 hr in the presence of IFN-γ (10 ng/mL) with either DMSO (0.15%) or Trikafta (5 µM). Ninety-six-well plates were centrifuged and supernatants were analyzed for murine TNF-α. Student’s *t*-test was performed for panels E–G and a two-way ANOVA with Sidak’s multiple comparisons was performed for panel H. For panels C–H, data points are three biological replicates, which represent the average of technical replicates.

Since Trikafta treatment takes place in the complex CF lung environment, we next explored how Trikafta co-treatment with stress agents and conditions found in the CF lung environment affects *A. fumigatus* biofilms. We co-treated 16-hour CEA10 biofilms with either DMSO or Trikafta with increasing doses of anti-fungal drugs, cell wall stress agents, metabolic stress agents, and oxidative stress inducers and measured overall metabolic activity as a readout of fungal cell damage using the XTT assay (27) (Fig. 6A and B). Trikafta alone did not affect metabolic activity of biofilms compared to DMSO controls after 3 hours of treatment (data not shown). Voriconazole, an anti-fungal drug targeting ergosterol biosynthesis, only mildly damaged the biofilms by about 20%–30%, as previously published (12). Intriguingly, Trikafta modestly protected biofilms from voriconazole-induced damage at this time point. Trikafta co-treatment with amphotericin B, which damages the cell membrane through direct binding of ergosterol, increased fungal cell damage. The effects of the echinocandin β-(1,3)-glucan synthase inhibitor, caspofungin, were exacerbated by Trikafta at a lower dose (0.015 µg/mL). Trikafta also exacerbated the effects of mitochondrial inhibitors FCCP [carbonyl cyanide 4-(trifluoromethoxy) phenylhydrazone] and antimycin A. Unexpectedly, Trikafta strongly protected biofilms from calcofluor white (CFW)-induced damage (Fig. 6A and B).

Since Trikafta had strong impacts on biofilm response to cell wall stressors CFW and caspofungin, we decided to further validate our screen results. We developed a modified checkerboard assay to test potential synergy/antagonism between Trikafta and caspofungin against *A. fumigatus* biofilms. We tested this by treating 16-hour biofilms with different dose combinations of caspofungin and Trikafta for 3 hours and measuring metabolic activity. We observed that at low doses of drug, Trikafta and caspofungin synergized with a score of 0.281 (Fig. 6C and D). Interestingly, we observed that at high doses of drug, Trikafta and caspofungin antagonized, with a score of 32 (Fig. 6C and D). To confirm if Trikafta and caspofungin synergize, we tested a low dose of Trikafta with a low dose of caspofungin on 16-hour biofilms and measured metabolic activity. We observed that Trikafta exacerbated a low dose of caspofungin-induced damage (Fig. 6E). We also observed that Trikafta protected biofilms from CFW-induced damage (Fig. 6F), demonstrating collectively that Trikafta alters biofilm responses to cell wall stressors.

Since Trikafta protected biofilms against CFW but exacerbated caspofungin effects, we hypothesized that Trikafta was likely increasing chitin and decreasing (1,3)-β-glucan content of the fungal cell wall. Furthermore, it has previously been shown that altering chitin content results in a compensatory effect that increases levels of (1,3)-β-glucan (28). Since mammalian cells recognize (1,3)-β-glucan, which triggers an inflammatory response, we hypothesized that Trikafta treatment would reduce the inflammatory response of host cells in co-culture with *A. fumigatus*. To test this hypothesis, CEA10 conidia were co-cultured with bone marrow (BM) cells from mice harboring the mouse dF508 mutation in both *cftr* alleles (29) for 16 hours. The supernatant was removed and assayed for tumor necrosis factor alpha (TNF-α) concentrations. Co-culture of murine bone marrow cells with *A. fumigatus* elicited an increase in TNF-α production; however, this effect was significantly reduced by Trikafta treatment (Fig. 6G). Finally, we tested whether Trikafta altered inflammatory cytokine production from WT BM cells. Intriguingly, Trikafta treatment also reduced TNF-α production from WT BM cells in co-culture with *A. fumigatus* compared to vehicle controls (Fig. 6H). Furthermore, A1160 and A1160-Δ*sakA* were equally susceptible to Trikafta-induced protection against the inflammatory response elicited in co-culture with murine BM cells, suggesting that the effects of Trikafta on the cell wall are upstream of SakA-mediated biofilm biomass reduction (Fig. 6H). These data collectively demonstrate that the effects of Trikafta alter the fungal cell wall, which subsequently change how host cells respond to *A. fumigatus*, independent of *cftr* mutations.

## DISCUSSION

The objective of this study was to determine if the current state-of-the-art treatment for pwCF, Trikafta, affects the biology of a common CF associated fungus, *A. fumigatus*. To begin to address this question, standard microbroth dilution MIC assays were utilized, and no anti-fungal effect was observed. However, the MIC assay tests the effects of a drug or small molecule against conidia, so we tested whether Trikafta affected *A. fumigatus* biofilms, which are found in an established infection. Unexpectedly, we observed that long-term (24-hour) treatment of biofilms with Trikafta reduced *A. fumigatus* biomass in several laboratory and clinical strains (Fig. 1D and E). The reduction in biofilm biomass correlated with reduced cell viability and increased membrane permeability (Fig. 2). Interestingly, the effects of Trikafta on permeability and viability occurred acutely, whereas biofilm biomass decrease was not observed until between 12 and 24 hours post treatment (Fig. 1B).

Surprisingly, there are only three major classes of approved anti-fungal drug therapies for the treatment of aspergillosis: polyenes, echinocandins, and azoles (30). Polyenes and azoles target the fungal cell membrane, and echinocandins target the fungal cell wall. Our data strongly suggest that Trikafta reduces viability in *A. fumigatus* biofilms by increasing membrane permeability and subsequent metabolic dysfunction. Within 1 hour of treatment with Trikafta, biofilms showed a significant increase in Sytox Blue staining and AK release (Fig. 2) and exacerbation of SDS-induced cell damage (Fig. 3D). These effects occur in parallel to Trikafta’s impact on overall viability of the biofilm. AK, which is highly expressed in the cytosol, is released extracellularly after the fungal cell integrity is compromised. It is also secreted when *A. fumigatus* is not actively growing or germinating (23). AK release has been successfully leveraged to identify anti-fungal compounds in several fungal species, such as *A. fumigatus* (23), *Candida albicans* (31), and *Cryptococcus neoformans* (32). Although Trikafta does not reduce overall growth of biofilms to the same magnitude as AmB treatment (Fig. 3C), we observed a striking effect of Trikafta on biofilm membrane permeability, viability, and growth. Considering the drug-resistant nature of fungal biofilms, future studies examining how these observations are relevant in the context of *in vivo*-relevant stressors are warranted.

Our data strongly suggest that Trikafta modulates ion channels in *A. fumigatus* biofilms. Given that treatment with Trikafta took more than 12 hours to affect biofilm growth, we were surprised to observe membrane permeabilization and reduced viability within 1 hour of treatment (Fig. 2A through C). Since we observed an effect on the cell membrane, we hypothesized that Trikafta’s mode of action is potentially like its function in mammalian cells. Previous studies in the literature have shown that calcium-activated chloride channels can also act as dual scramblases, which alter membrane fluidity and function (33, 34). Verapamil has been successfully utilized to block calcium channels in *Aspergillus fumigatus* (35), and our data demonstrate that it inhibits Trikafta-induced membrane permeability and biomass reduction (Fig. 3A and B). Our data also support the hypothesis that Trikafta may target multiple ion channels due to its exacerbation in conditions with high NaCl, CaCl_2_, and KCl levels (Fig. 3C). Since our null mutant for *cch1* is resistant to Trikafta damage in calcium stress, this lends support that there are multiple ion channel targets for *A. fumigatus*, particularly since the CEA10-Δ*cch1* strain is equally susceptible to Trikafta-induced biomass reduction as WT (Fig. 3E). We also searched for CFTR-like proteins using the human *cftr* sequence in the *A. fumigatus* genome and identified eight highly related sequences, with the top hit being AFUB_066250. This gene is a putative ABC multidrug transporter with orthologs shown to have roles in secondary metabolite biosynthetic processes. We generated a null mutant in this gene and did not observe differences in Trikafta-induced biomass reduction compared to WT and reconstituted controls (data not shown).

In addition to effects on membrane permeabilization and ion channel modulation, our data also suggest that Trikafta affects cell wall composition in fungal biofilms. Interestingly, when biofilms were exposed to Trikafta and lower concentrations of caspofungin, Trikafta increased biofilm susceptibility to caspofungin-induced damage at low doses of drug (Fig. 6A through E). Alternatively, Trikafta protected biofilms from high doses of caspofungin (Fig. 6A through D). When biofilms were exposed to Trikafta and the cell wall stressor, CFW, Trikafta protected the biofilms from CFW-induced damage (Fig. 6A through C). Since CFW directly binds and blocks chitin polymerization and higher doses of caspofungin induce chitin biosynthesis via the paradoxical effect (36), it is plausible that Trikafta increases chitin composition in the biofilm cell wall. The caspofungin paradoxical effect (CPE) occurs when (1,3)-β-glucan synthase is inhibited to such a degree that the cell compensates by increasing chitin content. CPE-induced chitin synthesis has been shown to be stimulated via the cell wall integrity pathway mitogen-activated protein kinase signaling cascade and the transcription factors RlmA and CrzA in *A. fumigatus* strain Af293 (37). However, this effect has been shown to be strain specific, as CrzA is not required for CPE in CEA10-derived strains (37). Furthermore, depletion of (1,3)-β-glucan synthases results in increased chitin in *Candida albicans*, which is dependent on calcium (38). Future studies examining the effects of calcium, CPE, and CWI pathway are critical in understanding how Trikafta affects cell wall composition.

Given our observations that Trikafta disrupts cell wall and cell membrane integrity, it is perhaps not surprising that we observed Trikafta-mediated phosphorylation of the *A. fumigatus* HOG pathway kinase SakA. However, two independent *sakA* null mutant strains were resistant to Trikafta-induced biomass reduction (Fig. 5A and B). Furthermore, *sakA* null mutant strains were not resistant to Trikafta effects on the inflammatory response (Fig. 6F), suggesting that the Trikafta/SakA phenotype is either downstream or independent of cell wall effects. One hypothesis we tested was that Trikafta induces an osmotic stress response in the biofilms; however, Trikafta did not increase glycerol accumulation, which is a classic response to osmotic stress (data not shown). In addition to the osmotic stress response, the HOG pathway has also been observed to be involved in general stress responses, such as those induced by oxidative stress. In *Aspergillus nidulans*, SakA has been shown to interact with other stress response proteins, such as SrkA, Mpkc, and AN6892 after treatment with hydrogen peroxide (39). This general stress response reduced mitochondrial function and caused cell cycle arrest. Furthermore, the SakA homolog Hog1 mutant has been shown to have increased respiration rates and altered mitochondrial membrane potential in *Candida albicans* (40). The widely used fungicide, fludioxonil, has been shown to cause fungal cell death by hyperactivating the Hog pathway (41, 42). In the context of Trikafta treatment of biofilms, increased ion channel activation could potentially cause SakA-dependent alterations in mitochondrial function and subsequent growth arrest, which was reduced in the biofilms lacking *sakA* (Fig. 5).

The fungal cell wall is critical in influencing the extent to which an immune response is generated against colonization or infection by fungal pathogens. The *A. fumigatus* cell wall contains galactomannan moieties as well as chitin and (1,3)-β-glucan, which all play a role in triggering an inflammatory response in the host [reviewed in reference (43)]. This is evidenced by early work showing that glucans can activate leukocytes, phagocytosis, and production of pro-inflammatory mediators (44). The C-type lectin receptor, Dectin-1, has been identified as the key host protein involved in recognition of fungal cell wall component (1,3)-β-glucan (45). Macrophages expressing high levels of Dectin-1 were shown to have an increased inflammatory response to *A. fumigatus*, which was completely blocked by neutralizing Dectin-1 antibodies (46). Furthermore, Dectin-1 can more efficiently recognize certain stages of *A. fumigatus* development that correspond with higher levels of β-glucans. Swollen conidia and germlings, which have the highest levels of β-glucan exposure, were shown to cause high levels of pro-inflammatory cytokine production (46). Our data demonstrate that Trikafta protects *A. fumigatus* biofilms from CFW (Fig. 6C) and causes increased susceptibility to caspofungin, which implies that there is less β-glucan content in the cell wall (Fig. 6D). This conclusion is supported by a decrease in TNF-α production in the supernatants of conidia and bone marrow cells in co-culture with Trikafta compared to vehicle controls (Fig. 6E and F). Since this phenotype is conserved in both WT and dF508 bone marrow cells, it is likely that this is attributed to the effect of Trikafta on the fungus itself, rather than host cells. It is also possible that this effect is due to non-CFTR-mediated effects (off-target) in the bone marrow cells. Further studies to determine how Trikafta affects the fungal cell wall and how this impacts host/pathogen interactions will be critical for determining ways to augment and/or leverage Trikafta’s antifungal activity in pwCF.

Our data suggest that the current CF treatment, Trikafta, causes biomass reduction, membrane permeabilization, and loss of viability in *A. fumigatus* biofilms. We observed that Trikafta alters *A. fumigatus* biofilm responses to cell wall stress, which correlated with reduced inflammatory cytokine production from bone marrow cells in co-culture with Trikafta-treated *A. fumigatus*. In our studies, we utilized drug concentrations that are potentially achievable in patients. U.S. Food and Drug Administration documentation showed C_max_ levels were 9.2 µg/mL for elexacaftor (we used 5 μM = 2.99 µg/mL), 7.7 µg/mL for tezacaftor (we used 5 μM = 2.60 µg/mL), and 1.2 µg/mL for Ivacaftor (we used 5μM = 1.96 µg/mL) in serum (47). To understand the implications of these observations more fully, *in vivo* models of allergic and invasive aspergillosis are necessary to define the effect of Trikafta on *A. fumigatus* biofilms, and the subsequent host response, in the mammalian lung.

## MATERIALS AND METHODS

### Strains and growth conditions

All strains utilized in this study are listed in Table 3. All strains were initially grown in agar (1.5%) plates containing 1% GMM (1% glucose, 6-g/L NaNO_3_, 0.52-g/L KCl, 0.52-g/L MgSO_4_•7H_2_O, 1.52-g/L KH_2_PO_4_ monobasic, 2.2-mg/L ZnSO_4_•7H_2_O, 1.1-mg/L H_3_BO_3_, 0.5-mg/L MnCl_2_•4H_2_O, 0.5-mg/L FeSO_4_•7H_2_O, 0.16-mg/L CoCl_2_•5H_2_O, 0.16-mg/L CuSO_4_•5H_2_O, 0.11-mg/L (NH_4_)_6_Mo_7_O_24_•4H_2_O, and 5-mg/L Na_4_EDTA; pH 6.5). Conidia were collected for experiments after growth at 37°C and 5% CO_2_ for 72 hours with 0.01% Tween-80 and filtered through miracloth (Millipore Sigma) to exclude hyphae.

**TABLE 3:** List of *A. fumigatus* strains used in the study

Strain	Origin or background strain	Source or genetic modification
Laboratory “wild-type” strains		
CEA10 (CBS144.89)	Patient with IPA^*a*^	CBS KNAW Fungal Biodiversity Centre
Af293	Lung biopsy: neutropenic patient	David Denning Laboratory
ATCC13073	Pulmonary lesion	American Type Culture Collection
Clinical isolates		
Af110-14.14	Sputum sample from pwCF	Dartmouth Hitchcock Medical Center
Af110-5.3	Sputum sample from pwCF	Dartmouth Hitchcock Medical Center
Af106-6.10	Sputum sample from pwCF	Dartmouth Hitchcock Medical Center
TP-9	Sputum sample from pwCF	Dartmouth Hitchcock Medical Center
Mutants		
A1160	CEA10	akuB^KU80^; pyrG (48)
Afs35	D141	akuA^KU70−^; ptrA (49)
CEA10-*gpdA*:GFP	CEA10	*gpdAp:GFP;hygB*
CEA10-Δ*cch1*	CEA10	*cch1* null mutant; Hyg (AFUB_010540)
CEA10-*cch1 ^rec^*	CEA10-Δ*cch1*	*cch1* reconstituted mutant at *atf4* save haven site; ptrA
A1160-Δ*sakA*	A1160	*sakA* null mutant; Hyg (AFUB_012420 (50),
Afs35-Δ*sakA*	Afs35	*sakA* null mutant; Hyg (51)
TP-9PBS2C1	TP-9	*pbs2* allele swap; ptrA (26)
TP-9PBS2C1: *sakA* Flag	TP-9	*pbs2* allele swap; *sakA*:Flag; Hyg (26)

^*a*^ IPA, invasive pulmonary aspergillosis.

### Biofilm growth and drug treatments

For all experiments, conidia from the indicated strains were counted and seeded in liquid GMM at 1.0 × 10^5^ conidia/mL for the indicated times in 6-well plates (for biomass assays) and 96-well plates for all other assays to develop immature biofilms. Media were then removed and replaced with GMM containing either DMSO or the indicated doses of ivacaftor (5 µM = 1.96 µg/mL), tezacaftor (5 µM = 2.60 µg/mL), elexacaftor (5 µM = 2.99 µg/mL), or the indicated combinations (Selleckchem). In ionic stress experiments, biofilms were grown in GMM, and at time of drug treatment, 500-mM ionic stressors were supplemented to the media. For drug co-treatments, all biofilms were grown as above. Biofilms were treated for 1 or 3 hours with the vehicle controls (DMSO or water), voriconazole (0.25- to 4.0-µg/mL DMSO, Sigma), amphotericin B (0.125- to 2.0-µg/mL DMSO, Cayman Chemicals), caspofungin (0.015625–4.0 µg/mL, Cayman Chemicals), calcofluor white (6.25–50.0 µg/mL, Sigma), GlyH-101 (20 µM, Selleckchem), verapamil (1 mM, Sigma), SDS (0.002%, Fisher), hydrogen peroxide (0.3125–5.0 mM, Sigma), FCCP (0.25–4.0 µM, Sigma), fludioxonil (31.25–500.0 µg/mL, Sigma), antimycin A (0.25–4.0 µg/mL, Sigma), and potassium cyanide (0.25–4.0 mM, Sigma).

### Quantification of submerged biofilm biomass

After growth in six-well plates and treatment for the indicated times, excess supernatant was removed from the biofilms by tilting plates on the side and removing pooled liquid. Biomass was then carefully scraped and collected into preweighed tubes. Samples were vortexed in 1-mL ddH_2_O and then centrifuged at 15,000 rcf for 10 min to pellet mycelia and then repeated. Supernatants were removed, frozen, then lyophilized. Dry biomass was then quantified.

### XTT/resazurin assay

After biofilm treatment with drugs or vehicle for the indicated times, media were removed. XTT solution (0.5-mg XTT/mL 1 × phosphate-buffered saline [PBS] with 25-µM menadione) (XTT sodium salt, VWR) was added at 150 µL per well and incubated at 37°C, 5% CO_2_, and 21% O_2_ until the positive vehicle control wells were reduced (1–2 hours). Next, 100 µL of the XTT solution supernatant was transferred to a 96-well plate, and optical density was measured at 450 nm. For resazurin experiments, a knockout library of putative *A. fumigatus* kinases was utilized (listed in Table 1) (50). Biofilms were grown to 16-hour maturity; media were removed; and fresh media with DMSO or Trikafta were added. After 30 minutes, media were supplemented with 10% resazurin and incubated for an additional 5 hours. Fluorescence at an excitation of 544 and an emission of 590 was used for quantification. To determine synergy/antagonism between Trikafta and caspofungin, a standard checkerboard assay (52) was modified for 16-hour biofilms. Trikafta working dose ranges used were 30.2 μg/mL (20 μM/molecule)–0.471875 μg/mL (0.3125 μM/molecule), and caspofungin working dose ranges were 8.0–0.015625 μg/mL in liquid GMM. Serial dilutions for Trikafta were inoculated in twofold dilutions vertically, and serial dilutions for caspofungin were inoculated in twofold dilutions horizontally. Media were removed from 16-hour biofilms, and media containing caspofungin/Trikafta dose combinations were transferred to biofilms for 3 hours. Media were then removed and XTT solution (described above) was added to biofilms for 1–2 hours, and the assay was performed as described above. The no-drug control well was set to 100%, and values of all other wells were calculated as a percentage of control. Fractional inhibitory concentration index was calculated based on the metabolic reduction concentration (MRC = less than 25% metabolic activity). Synergy/antagonism values were calculated as (MRC Drug A incombination with Drug B)/MRC Drug A + (MRC Drug B in combination with Drug A)/MRC Drug B. Any score less than 0.5 was considered synergy, and higher than 4 was antagonism.

### Adenylate kinase assay

To quantify alkaline phosphatase in biofilm supernatants, samples were assayed as previously described (23). Briefly, supernatants were collected and kept at 4°C for no longer than 24 hours. AK detection reagent (100-µL Toxilight non-destructive cytotoxicity bioassay, Lonza) was added to each well of samples (20 µL) and incubated at room temperature for 5 minutes prior to luminescence measurements (1-hour, 5-minute intervals).

### Sytox Blue stain of biofilms

To assess Sytox Blue staining in treated biofilms, biofilms were grown for 16 hours in filtered GMM; media were removed; and were treated as indicated. After treatment, biofilms were stained with Sytox Blue (Thermo Scientific) 1:1,000 for 5 minutes and then imaged using a Nikon spinning disc confocal microscope at ×20 magnification. Images were processed and analyzed using the Fiji image analysis software (53). Raw image stacks were resliced from top down without interpolation and maximum projected. The Fiji plugin JaCoP was used to analyze the co-localization of the cytosolic GFP signal with the Sytox signal (54). In the JaCoP plugin, the Sytox channel was set to channel 1, and GFP was set to channel 2. Channel thresholds were set manually to exclude the background signal. The Manders’ overlap was quantified between the two channels, and M2 was recorded as this represents the fraction of GFP-positive pixels that overlapped with Sytox-positive pixels as a readout for level of Sytox staining across conditions (55).

### Phospho-SakA Western blot

To assess phosphorylation of SakA by Western blot, a protocol for submerged biofilms was adapted from stationary cultures (26). Briefly, submerged biofilms (TP9^PBS2C1:*sakA*-Flag^) were grown in six-well plates for 16 hours; media were removed; and indicated treatments in GMM were added for an additional 30 minutes. Biomass was scraped into 2-mL screw cap tubes, frozen, and lyophilized. They were then bead beaten in 1-mL protein extraction buffer, and total protein was quantified by Bradford kit. For Western blotting, 40-µg protein was used. Total protein was transferred from a 10% SDS-PAGE gel onto a nitrocellulose membrane using the Trans-Blot turbo transfer system (Bio-Rad). Total Flag-SakA was detected using the Flag M2 antibody 1:2,000 (Sigma), and phosphorylated SakA was detected using anti-P-p38 antibody 1:1,000 (Cell Signaling). To quantify fluorescence, imaging was performed using the LI-COR Odyssey CLX System according to manufacturer’s protocols. Total Flag and phospho-SakA were normalized to total protein using the REVERT total protein stain (LI-COR Biosciences).

### Bone marrow co-culture

Bone marrow cells were collected from the tibia and femur of female C57Bl/6 WT and dF508 mice (10–12 weeks). The dF508 and littermate control breeders were obtained from Case Western Reserve University (Cystic Fibrosis Mouse Models Core). Bones were removed and BM cells were isolated by flushing bone marrow out using DMEM (Dulbecco's Modified Eagle's Medium)-based tissue culture media. Cells were pelleted; red blood cells were lysed; and cells were set up in co-culture with the indicated strains of conidia at an MOI (multiplicity of infection) of 1:10 supplemented with 20-ng/mL interferon gamma for 16 hours. Media were collected and assayed for total TNF-α concentration according to manufacturer’s instructions (R&D Systems).

### Statistics

All experiments were performed with at least three biological replicates, and data points were averages of technical replicates for each individual experiment. For experiments comparing two groups, a standard Student *t*-test was performed. For experiments comparing two groups under multiple conditions, a two-way analysis of variance (ANOVA) with Sidak’s multiple comparisons was used, or Dunnett’s multiple comparisons were used when comparing to one control. When comparing more than two groups, a one-way ANOVA with Tukey’s multiple comparisons was used. All statistical analyses were performed using GraphPad Prism version 9.4.1. For all graphs, ns (not significant), *P* > 0.05; *, *P* ≤ 0.05; **, *P* ≤ 0.01; ***, *P* ≤ 0.001, ****, *P* ≤ 0.0001. All diagrams were generated using BioRender.com.

## Data Availability

All underlying data are available from the corresponding author upon request.
